# Ionizing radiation induces epithelial–mesenchymal transition in human bronchial epithelial cells

**DOI:** 10.1042/BSR20200453

**Published:** 2020-08-07

**Authors:** Bo Tang, Yue Xi, Fengmei Cui, Jin Gao, Huiqin Chen, Wentao Yu, Yu Tu

**Affiliations:** 1 State Key Laboratory of Radiation Medicine and Protection, School of Radiation Medicine and Protection, Soochow University, Suzhou 215123, China; 2 Collaborative Innovation Center of Radiation Medicine of Jiangsu Higher Education Institutions, Suzhou 215123, China; 3 Department of Radiation Protection Safety, Shandong Center for Disease Control and Prevention, Jinan 250014, China

**Keywords:** 16HBE, EMT, FN1, ionizing radiation, RNA-seq

## Abstract

**Objective:** The present study aimed to analyze the mechanism by which long-term occupational exposure of workers to low-dose ionizing irradiation induces epithelial–mesenchymal transition (EMT) of the human bronchial epithelial cells using transcriptome profiling.

**Methods:** RNA-seq transcriptomics was used to determine gene expression in blood samples from radiation-exposed workers followed by bioinformatics analysis. Normal bronchial epithelial cells (16HBE) were irradiated for different durations and subjected to immunofluorescence, Western blotting, scratch healing, and adhesion assays to detect the progression of EMT and its underlying molecular mechanisms.

**Results:** Transcriptomics revealed that exposure to ionizing radiation led to changes in the expression of genes related to EMT, immune response, and migration. At increased cumulative doses, ionizing radiation-induced significant EMT, as evidenced by a gradual decrease in the expression of E-cadherin, increased vimentin, elevated migration ability, and decreased adhesion capability of 16HBE cells. The expression of fibronectin 1 (FN1) showed a gradual increase with the progression of EMT, and may be involved in EMT.

**Conclusion:** Ionizing radiation induces EMT. FN1 may be involved in the progression of EMT and could serve as a potential biomarker for this process.

## Introduction

People are inevitably exposed to ionizing irradiation in their daily lives and at work. The uncertainties about the health risks associated with long-term exposure to radiation at a low-dose in occupationally exposed workers have been the focus of research for many years. In their International Nuclear Workers Study (INWORKS), published in the International Journal of Epidemiology, in 2016, Hamra et al. specifically evaluated the radiation hazards and risk in a cohort of 600,000 nuclear workers in 15 countries [1]. Abbott A explored the risk of low-dose irradiation in 2015 [2]. Long-term general and occupational exposure to radiation has been the focus of research in the past. Keil et al. conducted a cohort mortality studies of underground miners from the Colorado Plateau [3]. Kamiya et al. conducted a cohort study of nuclear workers in U.S.A., and reported the ERR values of cancers and cardiovascular diseases in 2015 [4]. A recent epidemiological study emphasized the harmful effects of low-dose radiation exposure on human health and reported elevated cancer mortality among nuclear workers exposed to radiation at a cumulative dose <100 mSv and dose rate <10 mSv/year [5]. However, the biomarkers and molecular mechanism that predict the long-term and low-dose effect of radiation are not clear.

The carcinogenic effects of ionizing radiation rank first among other potential hazards of occupational exposure to radiation. It is of great clinical significance to detect and evaluate the carcinogenic role of radiation. In the early stages of exposure to radiation, several biological changes occur, of which epithelial–mesenchymal transition (EMT) is an important indicator of malignant change. EMT refers to the biological process in which epithelial cells are transformed into cells with interstitial phenotype characterized by loss of cell adhesion and enhanced ability to migrate [6]. Based on the specific biological environment in which MT occurs, there are three subtypes, of which type 3 EMT is associated with epithelial malignancies. Whether bronchial epithelial cells undergo EMT following long-term and low-dose radiation exposure and its mechanism are the focus of the present study.

The present study aimed to analyze the biological changes after exposure to long-term and low-dose ionizing radiation. High-throughput sequencing was used to analyze the mRNA expression profiles of workers with or without radiation exposure. Normal bronchial epithelial cells underwent EMT following multiple rounds radiation, and the differentially expressed mRNAs were validated using a cellular model. These findings provide an experimental basis for radiation-induced carcinogenesis.

## Subjects and methods

### Subjects

The present study was approved by the ethic committee of the Soochow University and informed consent was obtained from all participants. The study included a total of 22 participants as shown in Table 1, which included 10 workers with no radiation exposure and 12 workers exposed to radiation at a total dose of 63.3 mSv for an average of 9 years.

**Table 1:** General information pertaining to the participants

	Group	Cases	%
Age (year)	40–49	12	54.55%
	50–60	10	45.45%
Working age (year)	≤5	0	0.00%
	5–20	2	9.09%
	≥20	19	86.36%
Marital status	Married	22	100.00%
	Unmarried	0	0.00%
	Other	0	0.00%
Education	Elementary or less	0	0.00%
	Junior	4	18.18%
	Senior	7	31.82%
	Undergraduate or more	11	50.00%
Income per month (RMB)	<5000	18	81.82%
	≥5000	4	18.18%

### Reagent and cell line

Human normal bronchial epithelial cells (16HBE), culture medium, bronchial epithelial growth factor, and penicillin/streptomycin were purchased from Shanghai Zhongqiao Xinzhou Biological Company. Antibodies against FN1 (ab32419), vimentin (VIM, ab92547), E-cadherin (E-cad, ab40772), and the goat anti-rabbit IgG H&L (Alexa 594) secondary antibody (ab150080) were purchased from Abcam.

### Transcriptome sequencing

#### RNA library construction and sequencing

The ribosomal RNA (rRNA) was removed using the NEBNext rRNA Depletion Kit (New England Biolabs Inc., Massachusetts, U.S.A.). The RNA library was constructed using the NEBNext® Ultra™ II Directional RNA Library Prep Kit (New England Biolabs Inc., Massachusetts, U.S.A.). Quality control and quantification of the library were performed using a BioAnalyzer 2100 system (Agilent Technologies, U.S.A.) and sequenced using the 150 bp paired-end method in an Illumina Hiseq 4000. The reads were subject to quality control (Q30) and checked using the Cutadapt program (v1.9.3). The high-quality reads were mapped to the human reference genome (UCSC HG19) using the Hisat2 program (v2.0.4). Guided by the annotation from the TGF file, the Cuffdiff program was used to generate the transcript abundance of LncRNA and mRNA, known as FPKM (Fragments per kilobase of exon per million fragments mapped). The fold-changes between two groups were calculated and the differentially expressed mRNAs were identified based on *P*-value for analysis of GO and KEGG pathway.

#### GO and KEGG analysis

The Gene Ontology (GO) project (http://www.geneontology.org) provides a set of structured, controlled vocabularies for community use in annotating genes, gene products and sequences, which is divided by molecular function (MF), biological process (BP) and cell component (CC). GO terms with *P*-value ≤ 0.05 are considered to be statistically significant. By mapping multiple types of omics data, such as genomics, transcriptomics, proteomics, and metabolomics, into KEGG pathway, the biological function of these genes can be interpreted. *P*-value ≤ 0.05 was considered to be statistically significant.

### Real-time PCR

The plasma sample was thawed out from −70°C and centrifuged at 12,000 × ***g*** for 10 min at 4°C. Samples (250 μl) were transferred to another centrifuge tube (1.5 ml), and TRIzol LS Reagent (750 μl) and acetic acid (20 μl) were added. The homogenized samples were incubated with chloroform (0.2 ml). After shaking the tube manually for 15 s and incubating at 15 to 30°C for 2–3 min, the sample was centrifuged at 12,000 × ***g*** for 15 min at 4°C. Following centrifugation, the solution separated into a red phenol chloroform phase in the lower layer and a colorless aqueous phase in the upper layer. All the RNA were distributed in the aqueous phase. The volume of the aqueous phase was approximately 60% of the TRIzol LS Reagent added during homogenization. The aqueous phase was transferred to a new centrifuge tube and mixed with isopropanol for 10 min to precipitate the RNA. After centrifugation at 12,000 × ***g*** for 10 min at 4°C, the RNA became visible on the bottom and walls of the tube. The supernatant was removed and the RNA was washed with at least 1 ml of 75% ethanol. After centrifugation at 7,500 × ***g*** for 5 min at 4°C, the RNA was air-dried for 5–10 min. The concentration and purity of RNA were determined using a NanoDrop® ND-1000. RNA was reverse transcribed into cDNA using the SuperScript™ III Reverse Transcriptase (Invitrogen). Real-time PCR was performed using a qPCR SYBR Green master mix kit (CloudSeq), using the following condition: 95°C, 10 min; 40 cycles (95°C, 10 s; 60°C, 60 s). The melting curve was then obtained with the condition (95°C, 10 s; 60°C, 60 s; 95°C, 15 s) gradually from 60°C to 99°C (0.05°C/s). The relative expression of the genes were calculated using the 2^−△△CT^ method. The primers used are shown in Table 2.

**Table 2:** Primers used in the present study

Gene ID	Gene name	Primer	Sequence (5′–3′)
1	LAMB1	Forward	AAATCTTGTGCTTGCAATCCTC
		Reverse	CGCAAAGCAACTGTTGTTTAAG
2	KRT6B	Forward	GTACCAGACAAAGTACGAGGAG
		Reverse	GCTTGTTCTTAGCATCCTTGAG
3	FN1	Forward	AATAGATGCAACGATCAGGACA
		Reverse	GCAGGTTTCCTCGATTATCCTT
4	ADAM9	Forward	AGGCTGAAGGAAAAGAGCATAT
		Reverse	AAGTCCCTTCCTTGTTGTAAGT
5	RNF182	Forward	CCGTAGAGACAAAGCCGCC
		Reverse	TAAAGGCCACATGAAGGGTCT
Reference	GAPDH	Forward	GGCCTCCAAGGAGTAAGACC
		Reverse	AGGGGAGATTCAGTGTGGTG

### Cell culture and radiation

Human normal bronchial epithelial cells were purchased from Shanghai Zhongqiao Xinzhou Biological Company and cultured in medium supplemented with bronchial epithelial growth factor and penicillin/streptomycin (Sciencell, U.S.A.). When the cells were in logarithmic growth period, the culture dish was placed in a biological X-ray irradiator (RS-2000Pro) for radiation dose of 0.5 Gy at a dose rate of 1.11 Gy/min. After passage on the other day, the cells were exposed to radiation at the accumulative dose of 2 Gy. The radiation-exposed cells were divided into 0, 0.5, 1, 1.5, and 2 Gy groups.

### Immunofluorescence

Cells in the five groups were fixed with 4% paraformaldehyde (Meilunbio, China), cleared in 0.2% Triton X-100 (Meilunbio, China) for 10 min, and then blocked in the serum for 30 min. The cells were incubated with the primary antibody (Abcam, U.S.A.) at 4°C overnight and then incubated with the secondary antibody (Abcam, U.S.A.) at room temperature for 2 h in the dark. The nuclei were counterstained using DAPI and examined by fluorescence microscopy. Images were acquired using a scanning laser confocal microscope (FV1200), and the results were semi-quantitatively analyzed using the ImageJ software.

### Western blotting

Cells were lysed in lysis buffer containing PMSF (400 μl) on ice for 30 min, transferred to a centrifuge tube (1.5 ml) and centrifuged at 12,000 rpm for 5 min at 4°C. Protein concentration was determined using the BCA method (Solarbio, China) and the lysates were then subjected to sodium dodecyl sulfate polyacrylamide gel electrophoresis (SDS-PAGE). The proteins were transferred to polyvinylidene difluoride (PVDF) membrane and the membranes were blocked with 5% skim milk (Oxoid, U.K.) for 1 h. The membranes were then incubated with the primary antibody (Abcam, U.S.A.) at 4°C overnight and the secondary antibody (Abcam, U.S.A.) at room temperature for 1 h. The proteins were detected using enhanced chemiluminescence (ECL) immunoassay (Beyotime, China) and visualized in an AI680 ultra-sensitive multifunctional analyzer.

### Scratch assay

Cells in the five groups were plated in a six-well plate and cultured at 37°C. When the cells grew to 100% confluence, a uniform scratch was made in the cell monolayer using a 1 ml pipette tip. Then, the cells were washed with PBS and cultured with growth factor-free medium. Images were collected at 0, 8, and 20 h using a microscope (IX73), and were analyzed quantitatively using the ImageJ software.

### Adhesion assay

Cell adhesion was tested using a cell adhesion detection kit (BestBio, China) according to the manufacture’s protocol. Cells in the five groups were plated in 96-well plates at the density of 5 × 10^4^ cells/well in triplicates for each group. After 1 h of incubation at 37°C, the medium was changed and 10 μl of the staining solution B was added to the wells. The OD was measured at 450 nm after 1 h using a microplate reader (Synergy 2). The formula used for calculating the cell adhesion rate was: cell adhesion rate (%) = (OD of test cells − OD of the blank) / (OD of the control − OD of the blank) × 100

### CCK-8 assay

Cells in the five groups were plated in a 96-well plate at the density of 5 × 10^4^ per well in triplicates. On the next day, 10 μl of CCK-8 solution (Meilunbio, China) was added to each well, and the plates were incubated at 37°C. After 2 h, a microplate reader (Synergy 2) was used to determine the OD at 450 nm. The formula used for calculating cell viability was: cell viability (%) = (OD of test cells − OD of cell-free medium) / (OD of control cell − OD of cell-free medium) × 100.

### Statistical analysis

The general information of the participants was subjected to a normality test and expressed as mean ± standard deviation (SD). The data were analyzed using SPSS 20.0. For the quantitative data with normal distribution and homogeneity of variance, analysis of variance was used. For the qualitative data, chi-square tests were used. The significance level was set to *α* = 0.05 and *P*<0.05 was considered statistically significant.

## Results

### Baseline characteristics of participants

All the 22 participants were male. Except for salt intake, there were no significant differences among the other parameters, including age, smoking history, and eating habits (*P*<0.05) (Table 3).

**Table 3:** Baseline characteristics of participants

	Control	Radiation	*P*
Age	48.60 ± 4.79	50.50 ± 5.67	0.411
Smoke	50.00%	91.70%	0.056
Alcohol	50.00%	58.60%	0.696
Tea	70.00%	91.70%	0.293
Rice	10.00%	0	0.455
Vegetable	40.00%	66.70%	0.436
Oil	70.00%	58.30%	0.064
Sugar	20.00%	41.70%	0.424
Chili	20.00%	41.70%	0.485
Salted	50.00%	75.00%	0.440
Fruit	30.00%	66.70%	0.128
Salt	60.00%	33.30%	0.033
Meat	40.00%	66.70%	0.431

### Differentially expressed mRNA in radiation-exposed workers

**Figure 1:**
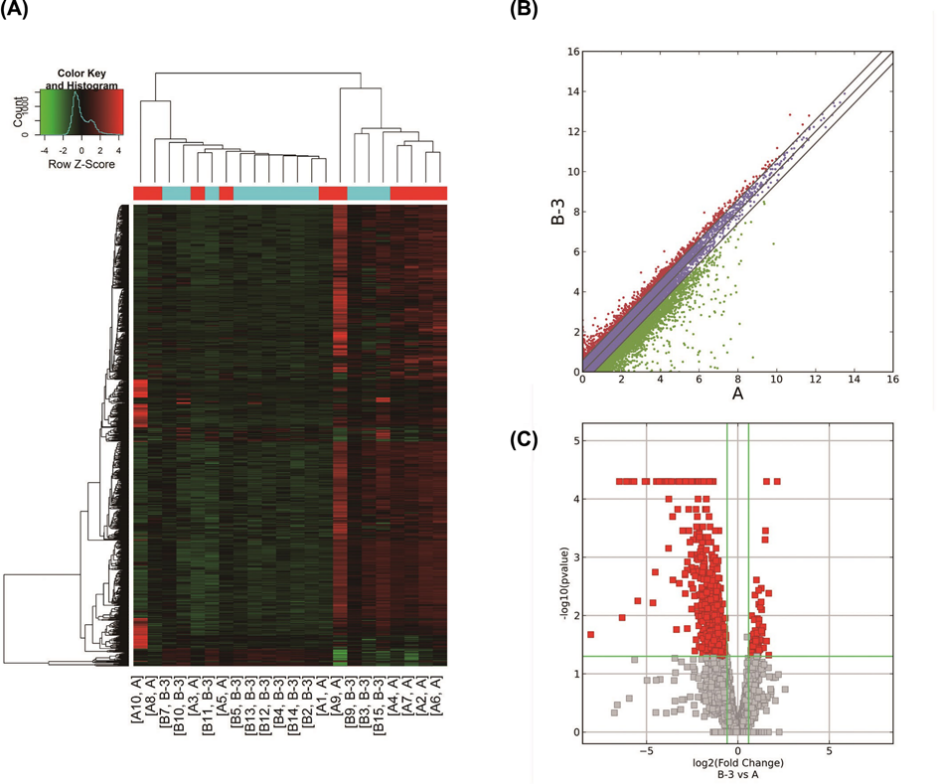
Differentially expressed mRNAs in workers with or without radiation exposure (**A**) Cluster analysis of differentially expressed mRNA based on FPKM values via heatmap2. In the picture, A is the control group and B is the exposed group. (**B**) Scatter plot based on the expression of the two groups. Red dots: up-regulated genes, green dots: down-regulated genes, purple dots: no significant change. Fold-change was 2.0. (**C**) Volcano plot based on fold-change and *P*-value. The red rectangle represents the differentially expressed mRNA, *P*≤0.05, fold-change ≥ 2.0.

The differentially expressed mRNA between the two groups were calculated using the Cuffdiff software with the fold-change ≥ 2.0, i.e., log (FC) ≥ 1.0, *P*-value ≤ 0.05, and FPKM value ≥ 0.5 in at least one sample as the threshold. Finally, we screened a total of 873 differentially expressed genes including 68 up-regulated and 805 down-regulated genes (Figure 1 and Supplementary Material S1).

### GO and KEGG analysis of differentially expressed mRNA

**Figure 2:**
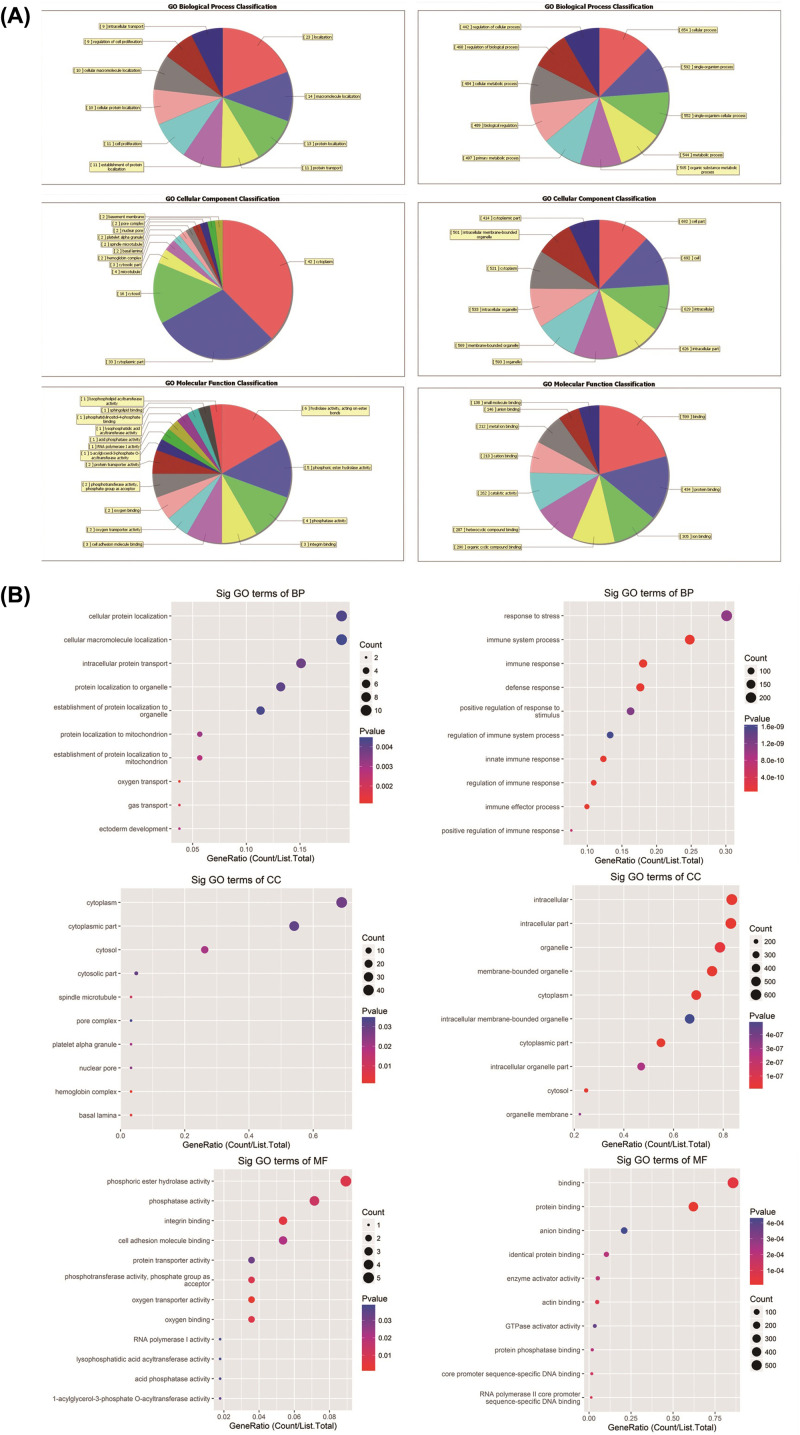
GO analysis of differentially expressed mRNA in workers with or without radiation exposure (**A**) The top 10 GO terms with the largest number of genes. (**B**) The ratio of the number of differentially expressed genes in the top 10 enriched GO terms with high fold enrichment to the total number of differentially expressed genes. The left column pertains to upregulated mRNAs, the right column pertains to down-regulated mRNAs, *P*≤0.05.

We then performed GO analysis on the differentially expressed mRNAs. For the up-regulated genes, there were 94 subclasses in BP, 18 in CC, and 16 in MF. For the down-regulated genes, there are 950 subclasses in BPs, 118 in CCs, and 113 in MFs (Figure 2). In BPs, the up-regulated mRNAs were involved in intracellular protein transport, cellular protein localization, ectoderm development, etc. In CCs, the up-regulated mRNAs were involved basal lamina, basement membrane, laminin complex, etc. In the MFs, the up-regulated mRNAs were involved in cell adhesion molecule binding and integral binding. For the down-regulated mRNA, the BPs mainly included immune response, immune system process, immune effector process, and defense response among others; the CCs mainly included membrane-bounded organelle, cytoplasm, and extracellular organelle; and the MFs mainly included protein binding, identification of protein binding, and cofactor binding among others.

**Figure 3:**
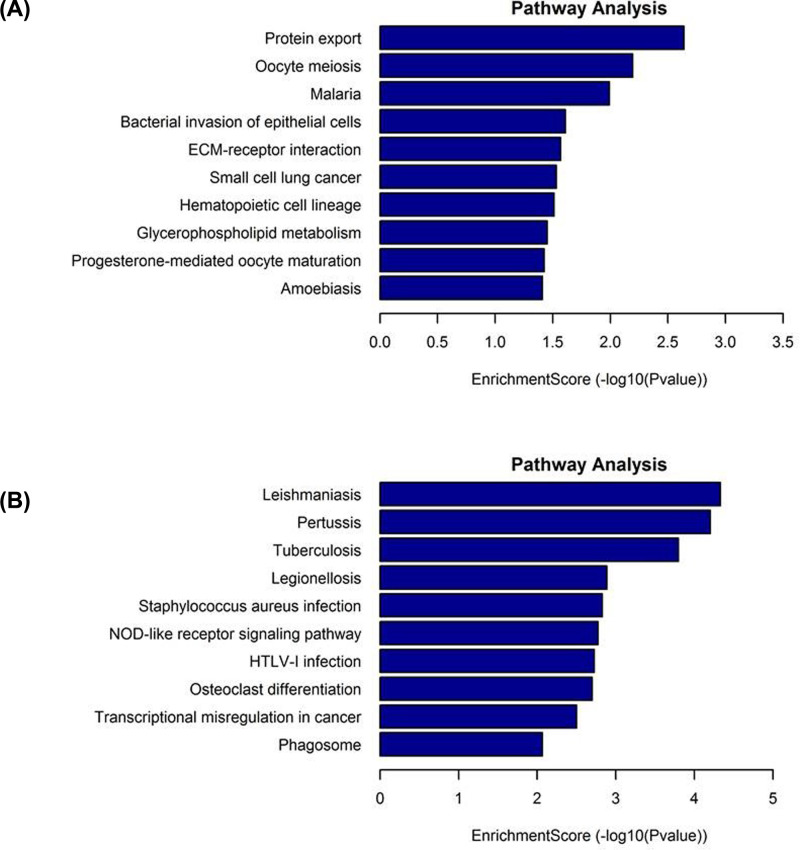
KEGG analysis of differentially expressed mRNA in workers with or without radiation exposure (**A**) The top 10 pathways with high enrichment scores associated with the up-regulated mRNA. (**B**) The top 10 pathways with high enrichment scores associated with the down-regulated mRNA.

KEGG analysis (Figure 3) revealed that 11 pathways were related to the up-regulated mRNA, mainly including protein export, bacterial invasion of epithelial cells, small cell lung cancer, and ECM–receptor interaction among others. There were 43 pathways associated with the down-regulated mRNA, mainly including pertussis and transcriptional mis-regulation in cancer.

Through the analysis of GO and KEGG, we found that the GO terms with higher enrichment and KEGG pathway were closely related to EMT, migration, and ECM–receptor interaction. Basement membrane, laminin complex, and cell adhesion molecule binding have been reported to participate in changes in cell morphology or cell migration. In summary, we believe that exposure to a certain dose of radiation may cause EMT or EMT-related regulatory processes.

### RT-PCR analysis of differentially expressed genes

**Figure 4:**
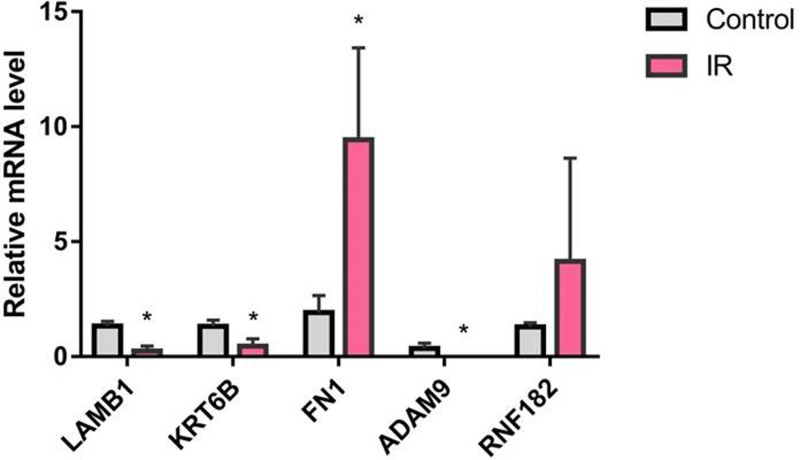
Validation of RNA-seq by RT-PCR *P<0.05 vs control.

GO and KEGG pathway analysis revealed that EMT-related regulatory processes, cell migration, and ECM–receptor interaction were closely associated with these differential expressed mRNAs. Based on these results, five candidate genes were selected, including LAMB1, KRT6B, FN1, ADAM9, and RNF182. RT-PCR analysis was performed to analyze the expression of these genes in the blood samples, which were also used for RNA-seq. The results showed that data from the RT-PCR assay were consistent with those from RNA-seq (Figure 4).

### Establishment of EMT model in 16HBE cells

**Figure 5:**
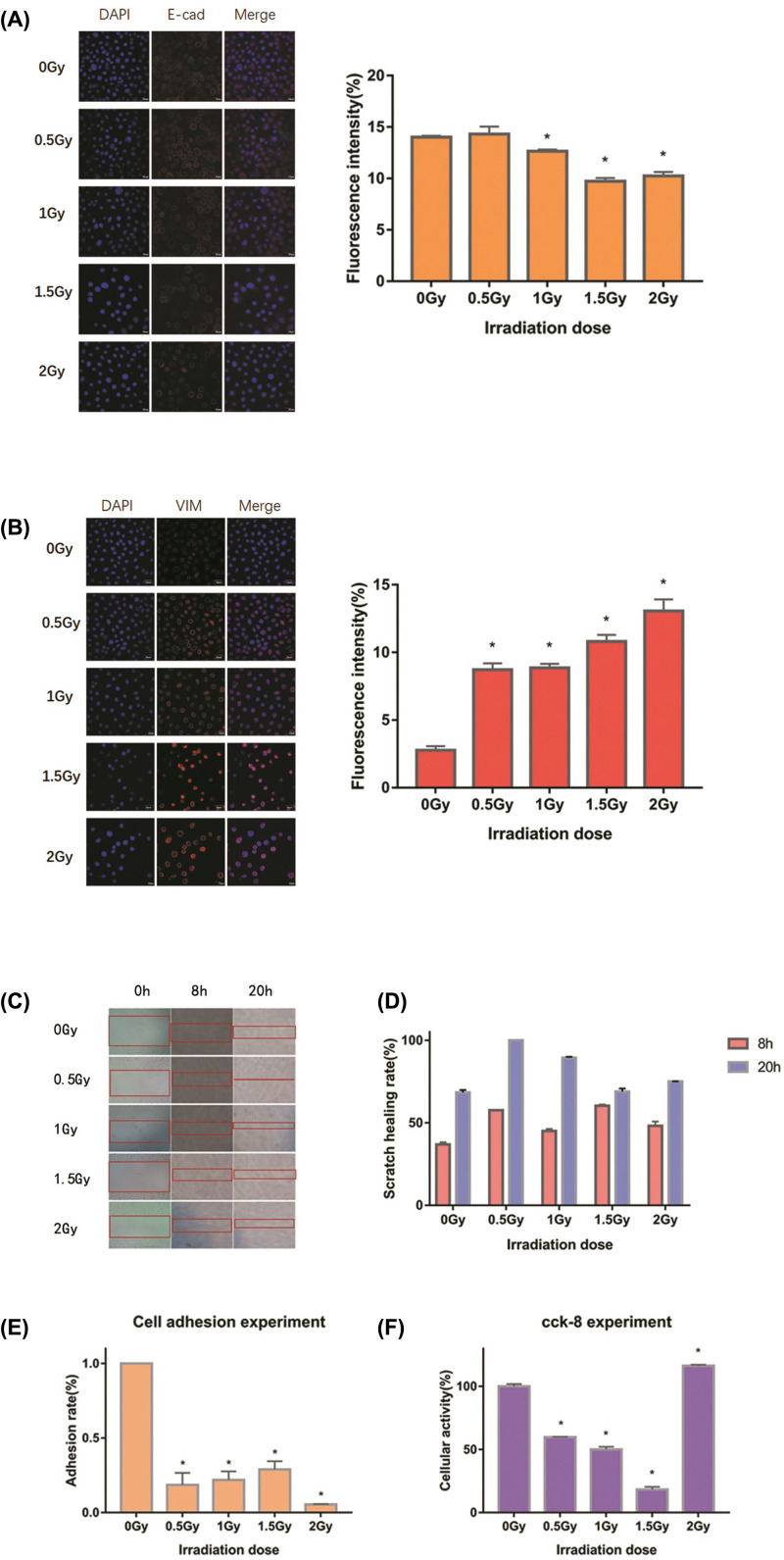
Changes in VIM, E-cad, migration, and proliferation of 16HBE cells following irradiation Images of E-cad (**A**) and VIM (**B**) expression at different cumulative doses of radiation observed under a confocal microscope. (**C** and **D**) Wound healing assay was performed to determine the cell migration capability. (**E** and **F**) Cell migration and activity were determined by adhesion and CCK-8 assays, respectively; *P<0.05 vs 0 Gy.

To further explore the function of FN1, we first observed the changes of EMT in human bronchial epithelial cells induced by long-term and low-dose radiation. As shown in Figure 5A,B, exposure to the cumulative dose of radiation resulted in a gradual decrease in the protein expression of E-cad and increase in VIM levels, indicating that these cells acquired EMT. The migration capacity and proliferation activity were also examined. Scratch healing assay revealed that the irradiated cells had a significantly higher migration capacity than normal 16HBE cells (Figure 5C,D). Cell adhesion assay showed that the adhesion rates of the irradiated cells were significantly lower than those without radiation exposure, which indirectly supported the observation that their migration ability was improved (Figure 5E). Moreover, CCK-8 assay show that the activity of the irradiated cells increased to a certain extent (Figure 5F).

### FN1 expression in 16HBE cells during EMT

**Figure 6:**
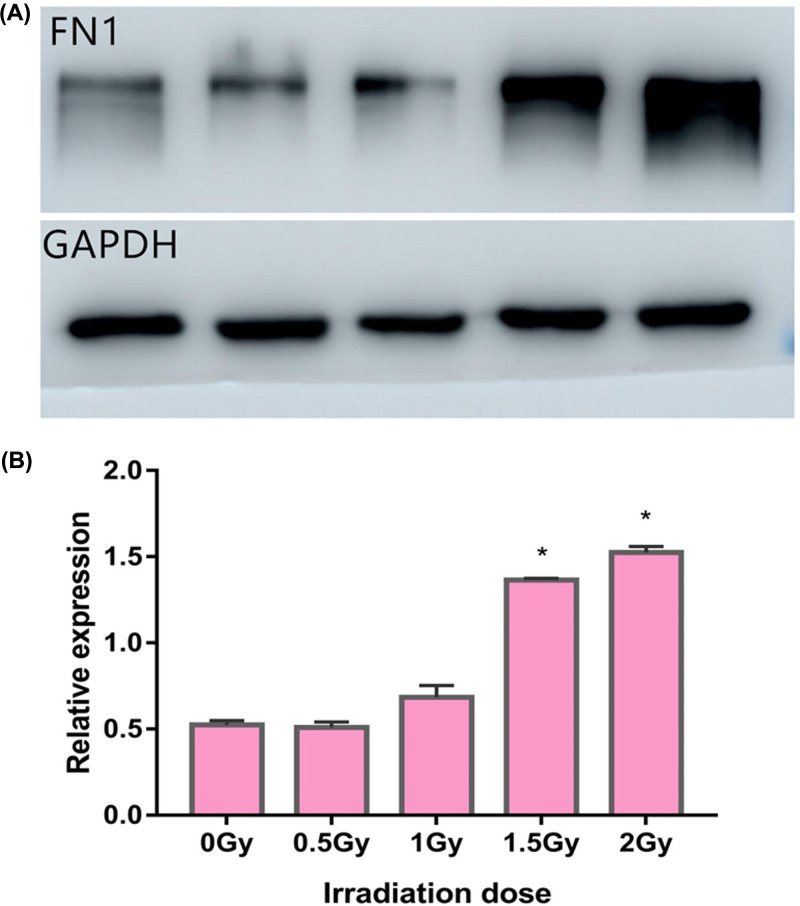
Expression of FN1 in 16HBE cells following different cumulative doses of radiation *P<0.05 vs 0vGy.

Western blotting was performed to analyze the expression of FN1 in 16HBE cells at different cumulative doses of radiation. As shown in Figure 6, the expression of FN1 gradually increased with increasing cumulative doses of radiation. The expression of FN1 was significantly increased at the radiation dose >1 Gy, showing that FN1 was involved in the EMT of 16HBE cells and may be used as a potential biomarker of the cumulative doses of radiation.

## Discussion

Lung cancer has always been an area of intensive research in the biological effects caused by ionizing radiation (and not just the epidemiological study of radiation) [7,8]. Owing to the occupational specificity and small sample size of the present study, for example, in some articles, the work content of many radiation workers involves confidentiality issues, which brings great difficulties to the pre-investigation work and the information of the personnel cannot be fully grasped, so the sample size is generally small. Studies using larger cohorts are still necessary to obtain more information, such as Mayak cohorts. The conclusions from these studies need further corroboration due to the differences of the research, applied and statistical methods used in the study [9–12]. In addition, gender-specific analysis was also limited due to the low number of female workers and the low exposure dose [13].

To identify radiation-related factors, high-throughput sequencing was performed using peripheral blood of healthy radiation-exposed workers. RNA-seq results revealed 873 differently expressed genes between participants with or without radiation. Among these genes, the up-regulated genes were mainly located on chromosome 2 and 7, while the down-regulated genes were in chromosome 1, 11, and 17. However, we did not identify any correlation between the distribution of chromosomal positions of these differential genes and ionizing radiation. Then, bioinformatics analyses (GO and KEGG) were conducted to identify the potential functions of these differential mRNAs. We found that the differential genes induced by radiation were mainly involved in the ECM–receptor interaction, laminin complex, cell adhesion molecule binding, and immune reaction. Based on these results, five differential genes were selected and validated using the same samples. Although there was no statistical difference in the smoking history between the two groups, smoking is a known high-risk factor for lung cancer, and cannot be eliminated as a factor. Studies have shown that smokers and non-smokers have different molecular mechanisms of lung cancer development, such as hypermethylation of gene promoters [14]. Therefore, the abnormal expression of some genes needs to be further studied to determine their correlation with ionizing radiation.

Among the up-regulated genes, SEC62, which is reported to be overexpressed in lung cancer, prostate cancer, and thyroid cancer [15], is involved in endoplasmic reticulum stress tolerance and cell migration, and is identified as a prognostic marker for non-small cell lung cancer [16]. Moreover, overexpression of SEC62 is also reported in the atypical fibrous xanthomas (AFX) [17]. CDC9, a member of tetraspanin superfamily, is involved in the regulation of many disease and physiological processes, including cell migration and adhesion [18,19]. PLD1 is the medium-membrane protein that is overexpressed in various cancers, such as lung cancer, breast cancer, and kidney cancer [20]. It also plays a key role in LA-induced breast cancer cell migration and invasion [21]. Thus, most of the up-regulated genes identified in our study are related to cancer.

Several prior studies used A549 as an *in vitro* model to study lung cancer [22–24], However, we used normal bronchial epithelial cells 16HBE in our study. EMT in 16HBE cells can be induced by silicon dioxide [25], smoking extract (CSE) [26], and transforming growth factor β1 (TGF-β1) [27–29]. The novelty of the present study was in the use of the 16HBE cells and the induction of EMT using ionizing radiation. Among the up-regulated genes, FN1 was found to be closely associated with EMT. Subsequently, RT-PCR and Western blotting further demonstrated that the expression of FN1 was increased in 16HBE cells following EMT. FN1, a member of the glycoprotein family, is involved in cell migration and adhesion [30]. Many studies have reported that FN1 has an indispensable role in EMT [31–34]. FN1, together with E-cad and VIM, serves as a marker for EMT in non-small cell lung cancer. Moreover, FN1 may play an important role in the pathogenesis of nasopharyngeal carcinoma [35] and the drug resistance of lung cancer [36].

In the present study, the mRNA expression of workers with or without radiation exposure was profiled using high-throughput sequencing and bioinformatics analysis. An EMT model of the normal bronchial epithelial cells was established after repeated irradiation of the cells. The differentially expressed mRNAs were selected based on the bioinformatics analyses and validated in the cellular model. We found that FN1 was involved in EMT and could be used as a potential biomarker. Our research provides evidence for the possible mechanism through which long-term exposure to ionizing radiation induces EMT.

## Abbreviations

16HBE: human normal bronchial epithelial cells
EMT: epithelial–mesenchymal transition
FN1: fibronectin 1
rRNA: ribosomal RNA
